# Long-distance continuous-variable quantum key distribution over 100-km fiber with local local oscillator

**DOI:** 10.1126/sciadv.adi9474

**Published:** 2024-01-03

**Authors:** Adnan A. E. Hajomer, Ivan Derkach, Nitin Jain, Hou-Man Chin, Ulrik L. Andersen, Tobias Gehring

**Affiliations:** ^1^ Center for Macroscopic Quantum States (bigQ), Department of Physics, Technical University of Denmark, 2800 Kongens Lyngby, Denmark.; ^2^ Department of Optics, Faculty of Science, Palacky University, 17. listopadu 12, 771 46 Olomouc, Czech Republic.; ^3^ Department of Photonics, Technical University of Denmark, 2800 Kongens Lyngby, Denmark.

## Abstract

Quantum key distribution (QKD) enables two remote parties to share encryption keys with security based on the laws of physics. Continuous-variable (CV) QKD with coherent states and coherent detection integrates well with existing telecommunication networks. Thus far, long-distance CV-QKD has only been demonstrated using a highly complex scheme where the local oscillator is transmitted, opening security loopholes for eavesdroppers and limiting potential applications. Here, we report a long-distance CV-QKD experiment with a locally generated local oscillator over a 100-kilometer fiber channel with a total loss of 15.4 decibels. This record-breaking distance is achieved by controlling the phase noise–induced excess noise through a machine learning framework for carrier recovery and optimizing the modulation variance. We implement the full CV-QKD protocol and demonstrate the generation of keys secure against collective attacks in the finite-size regime. Our results mark a substantial milestone for realizing CV quantum access networks with a high loss budget and pave the way for large-scale deployment of secure QKD.

## Teaser

Machine learning revolutionizes secure quantum communication, pushing its boundaries to unprecedented frontiers.

## INTRODUCTION

Secure exchange of cryptographic keys over public channels is a critical prerequisite for maintaining secure communication. Currently, this is often accomplished using public key cryptography based on computationally hard problems such as integer factorization and (elliptic curve) discrete logarithm, providing computational security (*1*, *2*). However, the emergence of advanced algorithms and quantum computers threatens the security of these methods (*3*, *4*). Quantum key distribution (QKD) offers a promising solution using the principles of quantum physics to share information-theoretically secure keys between remote users (*5*). However, the transmission range of QKD remains limited because of the inverse scaling of the secret key rate (SKR) with transmission distance (*6*), necessitating the use of trusted or untrusted nodes. Extending the distance between these nodes is vital for large-scale deployment of QKD.

While there have been numerous laboratory demonstrations and field trials for point-to-point long-distance QKD using discrete-variable protocols (*7*), continuous-variable (CV) encoding of quantum information, such as the amplitude and the phase quadrature of the electromagnetic field of light, offers a powerful approach for secure communication (*8*, *9*). This is because CV-QKD systems can be constructed using components found in coherent optical telecommunication systems, including in-phase and quadrature (IQ) modulators for quantum state preparation and coherent detection facilitated by a local oscillator (LO) for quantum state measurement. However, two major challenges in CV-QKD limit the transmission distance: excess noise (*10*), mainly originating from the laser’s phase noise, and limited classical information reconciliation efficiency (*11*).

To control the excess noise due to the laser phase noise, long-distance CV-QKD demonstrations usually transmit the LO (TLO) from the transmitter to the receiver (*12**–**16*). In such an implementation, the quantum state and the LO are prepared from the same laser source and propagate through an insecure quantum channel, ensuring a stable relative phase between the LO and the quantum signal. However, this configuration exposes the LO to potential adversaries, enabling side-channel attacks (*17*, *18*), and necessitates complex multiplexing techniques to avoid cross-talk from the strong TLO signal to the fragile quantum states (*19*).

CV-QKD systems with a locally generated LO at the receiver, also known as a real LO or local LO (LLO) configuration, can eliminate side-channel attacks on the LO and offer a practical and simplified optical subsystem (*20**–**24*). However, LLO CV-QKD suffers from high excess noise caused by phase noise originating from the utilization of two independent lasers, limiting its transmission distance (*25*).

Two recent LLO implementations (*26*, *27*) have claimed success in their attempts to demonstrate CV-QKD over a long distance of 100 km. In an endeavor to tackle the phase noise issue, Pi *et al.* (*26*) used an intricate system incorporating polarization multiplexing to separate a very strong pilot tone from the quantum signal, which makes their system suffer from some of the disadvantages similar to those that use a TLO. Moreover, in (*27*), the authors used an arbitrary postselection technique on data frames with low excess noise, leading to an underestimation of the excess noise and overestimation of the transmission range of the system. Last, none of these works considered finite-size effects (*28*), a crucial aspect for practical applications. Consequently, practical and long-distance LLO CV-QKD is yet to be achieved.

Here, we report to our knowledge the longest-distance experimental demonstration of LLO-based CV-QKD that implements the entire QKD protocol and generates keys while taking finite-size effects into account. Specifically, we achieved an SKR of 25.4 kbits/s over 100 km of ultralow-loss optical fiber with a total loss of 15.4 dB. This was made possible by controlling the excess noise using a machine learning (ML) framework for phase compensation (*23*) and optimizing the modulation variance for information reconciliation (efficiency β = 92.5%).

## RESULTS

### Residual phase noise

Excess noise in CV-QKD systems can arise from various sources, including quantization, modulation, relative intensity noise (RIN), Raman scattering, and residual phase noise (RPN). These noise sources are assumed to be statistically independent, and, therefore, the total excess noise can be expressed as the sum of individual contributions (*29*)

$$\xi =\xi_{\text{RIN}}+\xi_{\text{mod}}+\xi_{\text{quant}}+\xi_{\text{Ram}}+\xi_{\text{RPN}}+K$$ (1)

Among these noise sources, RPN, defined as the variance of the difference between the actual phase of the quantum signal and the estimated phase of the received signal, is the main source of excess noise in LLO CV-QKD. In the Gaussian-modulated coherent-state protocol, the excess noise due to the RPN at the receiver side can be expressed as (*25*)

$$\xi_{\text{RPN}}=2{\mathrm{TV}}_{\text{mod}}\left(\;1-e^{\frac{-V_{\text{RPN}}}{2}}\right)$$ (2)

where *T* is the transmittance, including the quantum channel and the detector efficiency; *V*_mod_ represents the modulation variance, i.e., the variance of the coherent-state ensemble; and *V*_RPN_ denotes the variance of the RPN.

Following Eq. 2, two options are available to reduce excess noise in LLO CV-QKD: operating the system at a low modulation variance or minimizing RPN. Although the former option is practical and straightforward to implement, it necessitates meticulous optimization of *V*_mod_ due to the dependence of the SKR on the modulation variance. In particular, both the mutual information and the efficiency of information reconciliation are influenced by the modulation variance.

Effective phase estimation is required to reduce RPN. Currently, the standard approach is to use pilot-aided techniques to estimate the relative phase between the free-running lasers of the transmitter and the receiver (*20**–**22*). The quality of the estimated phase depends heavily on the signal-to-noise ratio (SNR) of pilot-aiding signals, implemented using single-frequency tones or training symbols, transmitted together with the quantum signal. However, these methods are limited by channel loss, which increases with distance, and the need for a low-power pilot to reduce cross-talk to the quantum signal.

In contrast, ML has shown consistently excellent phase estimation performance across a wide range of pilot SNRs (*23*). This work combines ML-based phase estimation and modulation variance optimization to control excess noise to enable LLO CV-QKD over long distances.

### Data processing

**Fig. 1.**
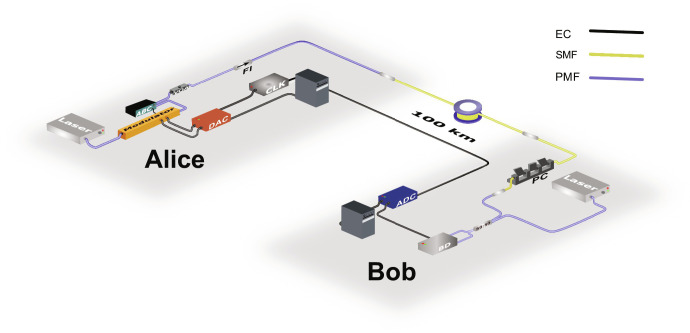
Long-distance continuous-variable–quantum key distribution (CV-QKD) system. Alice’s station consists of a continuous-wave (CW) laser operating at 1550 nm, an in-phase and quadrature (IQ) modulator with an automatic bias controller (ABC) for producing coherent states at sideband frequencies. A digital-to-analog converter (DAC) with a resolution of 16 bits and a sampling rate of 1 gigasample/s was used to drive the IQ modulator. A variable optical attenuator (VOA) was used after the IQ modulator to adjust the modulation variance of the quantum signal. A Faraday isolator (FI), whose forward direction is indicated by the arrow, is used before a 100-km ultralow-loss fiber channel that constitutes the quantum channel. Bob’s station consists of a polarization controller (PC) to adjust the polarization of the incoming signal and a balanced beam splitter to overlap this signal with a LO generated from another CW laser (unlocked/free-running with respect to Alice’s laser). The signal was detected and digitized using a balanced detector (BD), followed by an analog-to-digital converter (ADC) with a sampling rate of 1 gigasample/s. EC, electric connection; SMF, single-mode fiber; PMF, polarization-maintaining fiber.

**Fig. 2.**
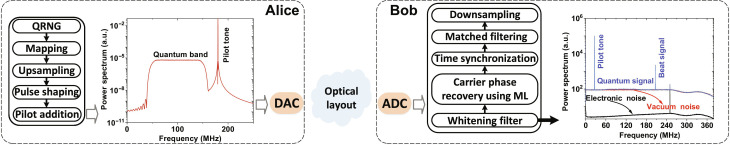
DSP routines of the long-distance local local oscillator (LLO) continuous-variable–quantum key distribution (CV-QKD) system. See Materials and Methods for the details. a.u., arbitrary units.

Figure 1 shows the schematic of the long-distance CV-QKD system, consisting of a sender (Alice) and a receiver (Bob) connected with a quantum channel made of 100-km ultralow-loss optical fiber. Alice prepared a 100-megabaud quantum signal in the single sideband of the optical carrier (*30*) together with a frequency multiplexed pilot, using a continuous-wave (CW) laser, an IQ modulator with automatic bias controller (ABC), and the digital signal processing (DSP) routine, shown in Fig. 2. Bob decoded the quantum information using radio frequency (RF) heterodyne detection and performed several DSP steps to recover a noisy version of Alice’s quantum symbols. These quantum data symbols were stored as frames by both parties for later offline data processing. Further details of the system implementation can be found in Materials and Methods.

After DSP, Alice and Bob perform data processing, including information reconciliation, parameter estimation, and privacy amplification (*30*). For information reconciliation, we used multidimensional reconciliation based on multiedge-type low-density parity- check (MET-LDPC) error-correcting codes with a rate of 0.05 (*31*). While this code was designed to ideally operate at a fixed SNR, in the experiment, the SNR can vary, e.g., because of polarization fluctuations. As a result, we achieved a reconciliation efficiency of β = 90.91% and a frame error rate (FER) of 0.0.

To enhance the reconciliation efficiency, we implemented a rate-adaptive reconciliation protocol that uses puncturing techniques to dynamically adjust the rate of the MET-LDPC code (*30**–**32*). With this technique, we achieved β = 92.5% at an FER of 0.59. While it is possible to achieve a higher efficiency of 93.1%, it comes at the expense of an increased FER of 0.80, which actually reduces the length of the final secret key (*32*). The reason behind the high cost of FER using the puncturing technique is that it increases the code rate by removing information. With less information, the probability that a frame is not decoded correctly increases, and, thus, it is expected that, with puncturing, the FER increases (*33*).

After error correction, parameter estimation was performed to evaluate the information advantage of the communicating parties over Eve by computing the Holevo bound. To accomplish this, we used all symbols, including the symbols of erroneous frames, i.e., the frames that Alice could not successfully decode. Last, we applied privacy amplification to generate the final key (*34*). The DSP and data processing were performed offline. For data processing, we developed a framework with a throughput of 5.9 megasymbols/s using the NVIDIA graphics processing unit (GeForce RTX 2060 Mobile) and a memory consumption of 2.5 gigabytes.

### Experimental investigation

**Fig. 3.**
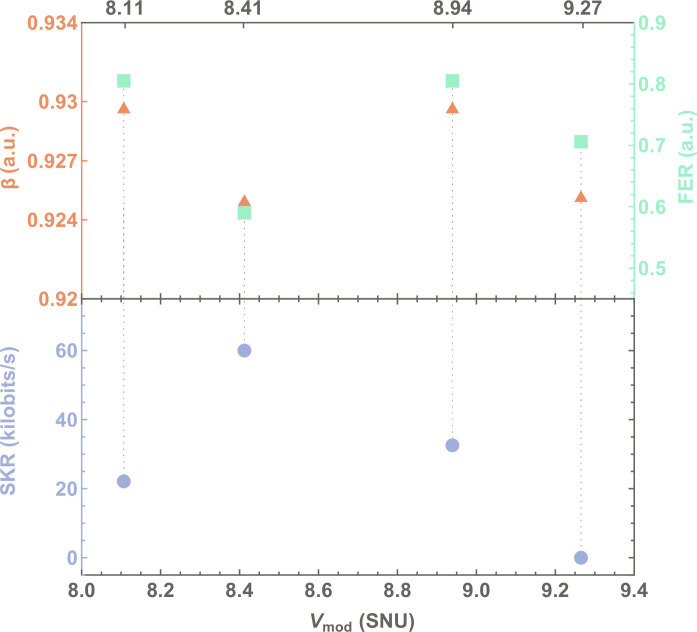
Modulation variance optimization. Experimentally obtained reconciliation efficiency β, frame error rate (FER), and asymptotic secret key rate (SKR) against the modulation variance *V*_mod_.

As the first step, we optimized the modulation variance for the given MET-LDPC code with a rate of 0.05. This was done by performing information reconciliation on four sets of measurements, each consisting of 10^8^ symbols, which were taken at different modulation variances. Figure 3 illustrates the figures of merit for information reconciliation (β and FER) and the overall system performance (SKR in the asymptotic regime) as a function of *V*_mod_. The highest efficiency of ≈93% with an FER of 0.8 was obtained at *V*_mod_ = 8*.*11 shot-noise units (SNU). However, this does not yield the best system performance in terms of SKR due to the high FER. Conversely, operating at the highest modulation variance of 9.27 SNU resulted in a lower FER of 0.7 but a null SKR due to the dominant effect of excess noise on system performance, as indicated by Eq. 2. Optimal system performance, striking a balance between β (92.5%) and FER (0.59) while maintaining low excess noise, was achieved by operating the system at *V*_mod_ = 8.41 SNU.

To evaluate the system performance, we used a security model with trusted devices (*28**,* *35*), assuming that some noise and loss are inaccessible to Eve. Table 1 summarizes the parameters used for secret key calculation. Alice generated an ensemble of 1 × 10^9^ coherent states at a symbol rate of 100 megabauds, with modulation variance of 8.41 SNU, and transmitted them through a quantum channel with a mean untrusted transmittance of η = 0*.*028 and a mean excess noise of ξ = 0*.*212 mSNU (at the channel output). This excess noise can be attributed to different sources with contributions as follows: RIN of the transmitter laser, ξ_RIN_ = 0*.*05 μSNU; digital-to-analog converter (DAC) noise, ξ_DAC_ = 1 μSNU; Raman noise from carrier and pilot tone, ξ_Ram_ = 7 × 10^*−*4^ μSNU; RPN after phase compensation, ξ_RPN_ = 160 μSNU; and other noise sources, ξ_other_ = 50*.*94 μSNU (see the Supplementary Materials for more details). The electronic/trusted noise of the detector and its efficiency/trusted transmittance had mean values of 62.72 mSNU and 0.68, respectively. For information reconciliation, Alice and Bob used 9*.*5 × 10^8^ symbols, with some symbols discarded because of time synchronization. With puncturing, we achieved an efficiency of 92.5% and an FER of 0.59.

**Table 1.** Final experimental parameters. τ, trusted efficiency; η, untrusted efficiency; *t*, trusted detection noise; ξ, excess noise; β, information reconciliation (IR) efficiency.

Alice	Bob	Channel	Infrared
*B* = 100 megabauds	τ = 0*.*68	η = 0.028	FER = 0*.*59
*V*_mod_ = 8*.*41 SNU	*t* = 62*.*72 mSNU	ξ = 0*.*212 mSNU	β = 92*.*5%

The key is deemed to be secure provided the positivity of the accessible information difference (*36*)

$$\begin{array}{c} \text{SKR}\left(\eta_{\text{low}},\xi_{\text{sup}}\right)=B\times \left(1-\mathrm{FER}\right)\left[\beta I_{\text{AB}}-\chi_{E}-\Delta \left(n\right)\right] \end{array}$$ (3)

where the mutual information between trusted parties *I*_AB_ and the upper bound on the information attainable by an eavesdropper χ_E_ are evaluated using η_low_ and ξ_up_ worst-case estimates of channel loss and excess noise, respectively, taken with error probability (i.e., the probability that true value falls outside the Gaussian confidence interval) of δ_fail_ = 10^*−*10^ (*37*). The correction term Δ(*n*) is related to the security of the privacy amplification procedure (*38*) on a key block of length *n* that is used to establish the key. In the asymptotic regime, the correction term Δ(*n*) is neglected, and true channel parameters η and ξ are presumed to be known and equal to estimated values.

**Fig. 4.**
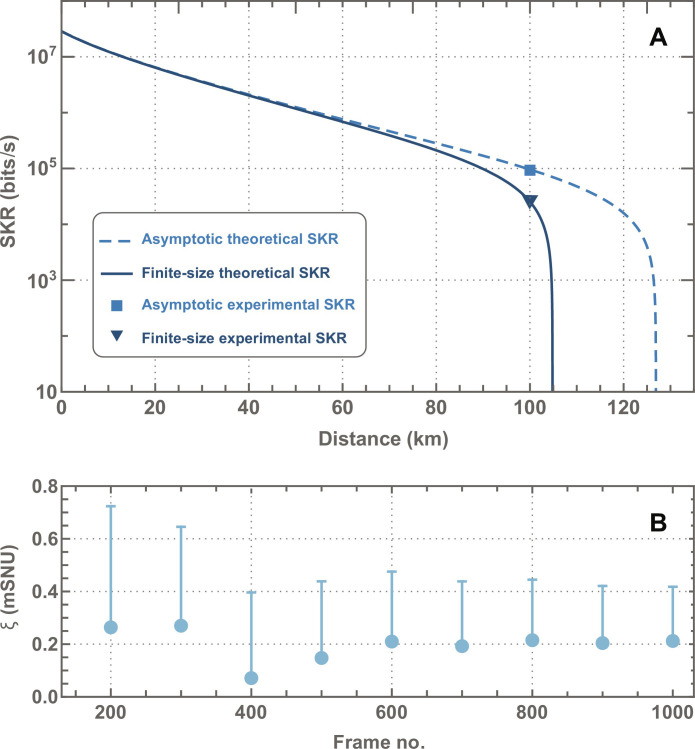
Performance of long-distance continuous-variable–quantum key distribution (CV-QKD). (**A**) The secret key rate (SKR) versus fiber channel length with an attenuation factor of 0.146 dB/km (taking into account additional coupling loss of 82%) in asymptotic (dashed) and finite-size (solid) regimes. Points correspond to experimentally achieved results. (**B**) Cumulative excess noise as a function of the number of acquired frames.

The SKRs for both numerical simulation and experimental results are depicted in Fig. 4A. The square and triangle correspond to our experimental results in asymptotic and finite-size regimes, respectively. A secure key generation rate exceeding 25 kilobits/s was achieved in both regimes. For given experimental parameters, one can theoretically predict a secure distance beyond 100 km.

Figure 4B shows the evolution of the excess noise with the frames accumulated and processed, with the respective upper bound of the confidence interval indicated by the dash. The protocol’s performance could be further improved by accumulating more data, consequently leading to tighter confidence in estimated channel noise.

## DISCUSSION

Long-distance transmission is a key requirement for large-scale deployment and integration of QKD in existing telecom networks. CV-QKD lends itself naturally to this integration. However, the secure and practical system configuration (LLO CV-QKD) faces limitations in transmission range due to the phase noise of lasers. In this work, we demonstrated long-distance LLO CV-QKD over a 100-km fiber channel, while accounting for finite-size effects. This record-setting experiment was made possible using ML for phase noise compensation and optimizing the modulation for information reconciliation and excess noise simultaneously.

Table 2 compares key aspects of long-distance CV-QKD experiments performed over the past 10 years. To achieve secure key generation beyond 70 km, previous demonstrations have used the TLO configuration and pulse carving, which introduce vulnerabilities and require an additional amplitude modulator, respectively. Thus far, the maximum distance of the LLO CV-QKD experiment with actual key generation was 60 km (*39*). However, this preliminary work presents a partial LLO CV-QKD system optimization compared with the present work. In particular, it only considered optimizing the modulation variance for phase noise. The recent demonstrations of 100-km LLO CV-QKD have not considered the finite-size regime, a crucial aspect for practical applications. The authors of (*27*) used an unjustifiable postselection technique on the frames with low excess noise, equivalent to underestimating the actual excess noise and overestimating the distance. Moreover, in (*26*), a DAC with only 8-bit resolution was used, indicating a poor approximation of the continuous Gaussian modulation. Furthermore, this work highlights the necessity of intricate system implementation, encompassing polarization multiplexing and an additional balanced detector, to segregate the strong pilot tone from the quantum signal effectively. Our work demonstrates the actual key generation in both asymptotic and finite-size regimes considering collective attacks. This achievement closes the gap between LLO-CV-QKD and TLO-CV-QKD systems’ performance while maintaining a high level of security and lowering the implementation complexity.

**Table 2.** Comparison of long-distance continuous-variable–quantum key distribution (CV-QKD) demonstrations.

Reference	Laser source	LO	Distance	Loss	Modulation	Security
(*13*)	Pulsed	TLO	80 km	16.1 dB	Gaussian	Finite-size
(*15*)	Pulsed	TLO	100 km	20 dB	Gaussian	Finite-size
(*16*)	Pulsed	TLO	202.18 km	32.45 dB	Gaussian	Finite-size
(*39*)	CW	LLO	60 km	13.09 dB	Gaussian	Asymptotic
(*26*)	CW	LLO	100 km	–	Gaussian	Asymptotic
(*27*)	CW	LLO	100 km	18.96 dB	Gaussian	Asymptotic
Current work	CW	LLO	100 km	15.4 dB	Gaussian	Finite-size

Nonetheless, there is substantial room for improvement in the current implementation. The overall system performance can be improved using MET-LDPC with a more suitable code rate of 0.03, allowing the system to operate at the optimal modulation variance of ≈3.5 SNU for β = 92%. This can lead to over a fivefold increase in the key rate, and even more notable improvement with higher error correction efficiencies. To achieve composable security *(**30*), under current parameters, collecting a large number of symbols (≈10^12^) remains crucial, as described in more detail in the Supplementary Materials. However, improving the reconciliation (β → 96% and FER → 10%) while maintaining optimal modulation variance can substantially relax the block size requirement (≈2 × 10^10^). An additional improvement avenue involves increasing the symbol rate of the system. This can be achieved by increasing the system’s bandwidth, for instance, using high-speed DACs and analog-to-digital converters (ADCs) combined with a broadband balanced detector (*40*). Besides, for real-time implementation, online data processing using graphics processing unit–based implementation can be considered (*41*).

In summary, this experiment has the potential to pave the way for realizing CV quantum networks, such as quantum passive optical networks, where high loss tolerance and LLO are essential ingredients. We believe that this will ultimately be a key enabler for the large-scale deployment of secure quantum communication.

## MATERIALS AND METHODS

### Details of the experimental setup

#### Optical layout

Figure 1 shows the optical layout of our long-distance LLO CV-QKD system based on the Gaussian-modulated coherent-state protocol. At the sender, Alice, a CW laser with a narrow line width of ≈100 Hz and operating at a wavelength of 1550 nm was used as an optical carrier. The coherent states were prepared by modulating the CW laser using an IQ modulator driven by a 16-bit DAC with two channels operating at a sampling rate of 1 gigasample/s. The IQ modulator was operated in single sideband mode by controlling the direct current bias voltages using an ABC. A variable optical attenuator (VOA) was placed after the IQ modulator to adjust the modulation variance of the thermal state. A Faraday isolator was added at the sender output to avoid any back-reflections from the channel and Trojan horse attacks. The signal was sent through a quantum channel made of a commercial ultralow-loss fiber (TeraWave SCUBA 150 Ocean Optical Fiber). The fiber attenuation is 0.146 dB/km at 1550 nm. The total loss in our 100-km fiber channel was 15.4 dB due to the mode field diameter difference between the SMF28 fiber pigtail and SCUBA 150.

At the receiver, Bob, RF heterodyne detection was used for the quantum state measurement. To accomplish this, another CW laser, free-running with respect to Alice’s laser, was used as the LLO. The frequency difference between Alice’s and Bob’s lasers was ≈230 MHz. The polarization of the quantum signal was then tuned to match the polarization of the LLO using a polarization controller. Next, the quantum signal and the LLO were combined on a balanced beam splitter, followed by a home-made balanced detector with a bandwidth of ≈365 MHz to detect the interference pattern. Last, the detected signal was digitized using a 16-bit ADC with a sampling rate of 1 gigasample/s and recorded for offline DSP. The ADC and DAC were synchronized using a 10-MHz reference clock (CLK).

The measurement time was divided into frames, each containing 10^7^ ADC samples. Using a framework based on Python, three measurements were taken consecutively without user intervention: quantum signal measurement, vacuum noise measurement (Alice’s laser off and Bob’s laser on), and electronic noise measurement (Alice’s laser off and Bob’s laser off). The framework minimized the latency between these measurements. We determined that our system is stable for at least 25 s as detailed in the Supplementary Materials, where more information about the system stability can also be found. The clearance of the vacuum noise over the electronic noise was ≈15 dB in the frequency band of the quantum signal. To calibrate the *V*_mod_ of the thermal state, we performed back-to-back measurements, in which Alice and Bob were connected through a short fiber patch cord, and the VOA was finely tuned to set different *V*_mod_ values.

#### Digital signal processing

Figure 2 shows the offline DSP routine used for digital waveform generation and quantum symbols recovery at Alice’s and Bob’s stations, respectively. To produce an ensemble of coherent states, a sequence of random numbers with Gaussian distribution was generated by mapping the uniformly distributed output of a quantum number generator based on vacuum fluctuation (*42*). These numbers form the complex amplitudes of the quantum symbol α*_i_* = *x_i_* + *ip_i_* used for IQ modulation. These quantum symbols were drawn at a symbol rate of 100 megabauds, upsampled to 1 gigasample/s and pulse-shaped using a root-raised cosine filter with a roll-off factor of 0.2. For single-sideband modulation, the quantum signal was frequency shifted to 100 MHz. To this passband quantum signal, a pilot tone was multiplexed in frequency at 180 MHz for frequency and phase estimation at the receiver. The spectrum of the generated digital waveform is shown in the left part of Fig. 2. Last, Alice uploaded her waveform into the DAC to obtain the corresponding electrical analog signals.

Extensive frame-to-frame DSP was deployed to reconstruct the quantum symbols at the receiver. After the digitization process using the ADC, a frequency domain equalizer (whitening filter) was applied to the quantum signal, vacuum noise, and electronic noise measurements. The filter coefficients were computed by taking the inverse of the receiver frequency response and averaging over 1000 frames. This step is essential to remove any autocorrelation and thus preserve the condition of independent and identically distributed quantum symbols. The whitened spectrum of the quantum signal, vacuum noise, and electronic noise is shown on the right side of Fig. 2. To estimate the phase and frequency difference between Alice’s and Bob’s lasers, a bandpass filter of 1 MHz was used to extract the desired pilot tone, with an SNR of ≈23 dB. The phase profile was extracted by computing a Hilbert transform of the filtered pilot. The frequency offset was then estimated using a linear fit. Using the estimated frequency offset, the pilot tone was baseband-transformed and then used as an input signal to an ML framework based on an unscented Kalman filter for phase estimation (*23*). After phase estimation, the quantum signal was shifted to baseband using the pilot frequency estimate and the known frequency offset between the quantum signal and pilot tone. The estimated phase from the unscented Kalman filter was used for correcting the phase of the quantum signal. The cross-correlation between reference transmitted samples and the receiver samples was used to compensate for the propagation delay of the fiber channel and different electronic components. Then, the quantum symbols were recovered after matched root-raised cosine filtering and downsampling. Last, Bob compensated for the slow phase drift (θ), resulting from the frequency difference between the pilot tone and the quantum signal, using some reference symbols to maximize the covariance as, $\underset{\theta }{\text{argmax}}\text{Cov}\left(\gamma \text{exp}\left(j\theta \right),\alpha \right)$ , where γ and α are a known subset of Bob’s symbols and the reference symbols, respectively.

## Supplementary Materials

### This PDF file includes:

Supplementary Text Figs. S1 to S6 References
