# Supplementary material for: Long-distance continuous-variable quantum key distribution over 100-km fiber with local local oscillator
Source: Sci Adv. 2024 Jan 3;10(1):eadi9474. doi: 10.1126/sciadv.adi9474 (PMC10776027; doi:10.1126/sciadv.adi9474)
Supplement: Supplementary file 1 — Supplementary Text Figs. S1 to S6 References [file sciadv.adi9474_sm.pdf]

Science Advances
AAAS

Supplementary Materials for

Long-distance continuous-variable quantum key distribution over 100-km fiber with local local oscillator

Adnan A. E. Hajomer *et al.*

Corresponding author: Adnan A. E. Hajomer aaeha@dtu.dk; Tobias Gehring tobias.gehring@fysik.dtu.dk

Sci. Adv. **10**, eadi9474 (2024)
DOI: 10.1126/sciadv.adi9474

This PDF file includes:

Supplementary Text
Figs. S1 to S6
References

S1. Excess noise contributions

In this section we break down the total excess noise into contribution from some known sources to get a better understanding of the limitations.

Relative intensity noise The transmitter laser has intensity noise which can impact state preparation. To understand how to estimate the excess noise due to the relative intensity noise (RIN) of Alice's laser, Fig. S1 shows a simple sketch of the transmitter and the spectra at different points. To generate the quantum signal, the IQ modulator displaces the quantum state in the sidebands of the optical carrier, which given the existence of RIN in the laser, can be assumed to be a thermal state without loss of generality. To compute the excess noise ξ_{RIN} caused by the RIN the idea is to start at the RIN value given in the datasheet of the laser (NKT Photonics Basik E15), specified as RIN $= -135$ dBc/Hz at 10 MHz sideband and 1 mW carrier power, from which we compute the thermal state variance and then the excess noise at channel output.

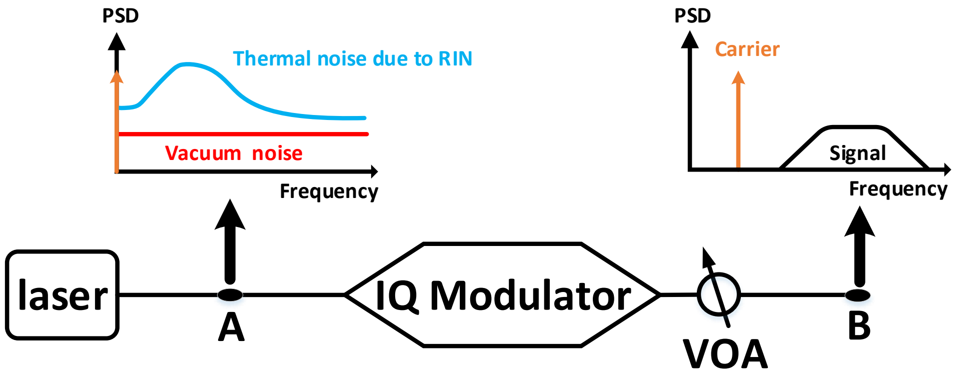

Fig. S1. Block diagram for characterizing RIN's excess noise. VOA: variable optical attenuator.

We start with single-sided power spectral density (PSD) estimates obtained through 'fictitious' measurements using a photodetector at point **A** in Fig. S1 with P^{A}_{carrier} denoting the carrier power. Given a photocurrent I, the (linearized) RIN and the PSD are related by

$$\mathrm{PSD}_I(f) = \mathrm{RIN}_I(f, P^{A}_{\mathrm{carrier}}) \times (P^{A}_{\mathrm{carrier}}) \ , \tag{S1}$$

while the PSD of the vacuum noise is a constant given by

$$\mathrm{PSD}_{\mathrm{vacuum}} = 2h\nu \ . \tag{S2}$$

where h is Planck's constant and ν is the frequency of the laser. Evaluating the two expressions at 1 mW carrier power ($P^{A}_{\mathrm{carrier}} = 1$ mW) and assuming the constant RIN specified in the datasheet then yields the normalized thermal state variance $\tilde{V}_{\mathrm{thermal}}$ by taking the ratio of the evaluated PSDs, i.e.,

$$\tilde{V}_{\mathrm{thermal}} = 10^{[\mathrm{RIN}+10\log(1\,\mathrm{mW})-10\log(2h\nu)]/10} . \tag{S3}$$

Finally, we attenuate the beam with $\zeta = \frac{P^{B}_{\mathrm{carrier}}}{1\,\mathrm{mW}}$, where P^{B}_{carrier} is the carrier power at point **B** of Fig. S1 (i.e., after the carrier suppression from the IQ modulator and attenuation applied by the VOA).

We are left with experimentally determining the optical carrier power leaving the transmitter, which we do using the measurement over the 100 km distance. From the measurement, we determine the power by

$$P^{B}_{\text{carrier}} = h\nu \frac{\text{PS}_{\text{beat}}}{T\ \text{S}_{\text{vacuum}}}, \tag{S4}$$

where PS_{beat} is the power spectrum of the beat signal at the receiver, S_{vacuum} is the PSD estimate of the vacuum noise evaluated (using analog-to-digital converter (ADC) samples) at the beat frequency and T is the total transmittance. By substituting in Eq. S4 $T = 0.019$, $\text{PS}_{\text{beat}} = 4.269 \times 10^3$ a.u and $\text{S}_{\text{vacuum}} = 1.229 \times 10^{-3}$ a.u/Hz, which are computed from the experimental measurement, we obtain $P^{B}_{\text{carrier}} \approx 23.42$ pW, and therefore, $\zeta = 2.342 \times 10^{-8}$. Finally, we can compute ξ_{RIN} at the receiver side as,

$$\xi_{\text{RIN}} = T\zeta(\tilde{V}_{\text{thermal}} - 1), \tag{S5}$$

which is ≈ 0.05 μSNU.

The calculation above assumed symmetric interferometer arms of the modulator so that the sideband noise suppression is identical to the carrier suppression. Furthermore, we assumed that the RIN itself is not modulated. To demonstrate that, indeed, this contribution is small, we extend our analysis in the following with a complete quantum mechanical description.

Figure S2 shows the schematic of the IQ modulator, consisting of nested Mach-Zehnder modulators (MZMs) modulating the amplitude and the phase quadrature. These MZMs are presumed to be biased at θ_1 and θ_2 and are driven by signals of the form $m\cos\Omega t$ and $m\sin\Omega t$ for the purpose of single sideband modulation where m is a function of time for broadband quantum signals and assumed to be small with respect to the half-wave voltage of the modulator. For a given input $\gamma + \hat{\delta} b$, where γ represents the carrier component and $\hat{\delta} b$ denotes a thermal mode and vacuum modes $\hat{\delta}\nu_x$, the output of the IQ modulator can be written as,

$$\hat{C} = \frac{1}{\sqrt{2}}\left(\hat{B}e^{i\phi} - \hat{A}\right), \tag{S6}$$

where

$$\hat{A} = \frac{1}{2}\left\{\hat{I}\left(e^{i\theta_1} + e^{im\sin\Omega t}\right) + \hat{\delta\nu_1}\left(e^{i\theta_1} - e^{im\sin\Omega t}\right)\right\}, \tag{S7}$$

$$\hat{B} = \frac{1}{2}\left\{\hat{Q}\left(e^{i\theta_2} + e^{im\cos\Omega t}\right) + \hat{\delta}\nu_2\left(e^{i\theta_2} - e^{im\cos\Omega t}\right)\right\}, \tag{S8}$$

$$\hat{I} = \frac{1}{\sqrt{2}}\left(\gamma + \hat{\delta} b - \hat{\delta}\nu_g\right), \hat{Q} = \frac{1}{\sqrt{2}}\left(\gamma + \hat{\delta} b + \hat{\delta}\nu_g\right). \tag{S9}$$

The condition for obtaining perfect single sideband suppression is $\phi = \pi/2$. Assuming that and employing the Jacobi–Anger expansion, we can expand the output of the IQ modulator as follows,

$$\begin{aligned}
\hat{C} \approx& \frac{1}{2\sqrt{2}}\Bigg[\left(\hat{Q}\left(e^{i\theta_2} + J_0(m) + 2iJ_1(m)\cos\Omega t\right) + \hat{\delta}\nu_2\left(e^{i\theta_2} - J_0(m) - 2iJ_1(m)\cos\Omega t\right)\right)i - \\
&\hat{I}\left(e^{i\theta_1} + J_0(m) + J_1(m)e^{i\Omega t} - J_1(m)e^{-i\Omega t}\right) - \hat{\delta}\nu_1\left(e^{i\theta_1} - J_0(m) - J_1(m)e^{i\Omega t} + J_1(m)e^{-i\Omega t}\right)\Bigg] \\
\approx& \frac{1}{2\sqrt{2}}\Bigg[\left(\hat{Q}\left(e^{i\theta_2} + J_0(m)\right) + \hat{\delta}\nu_2\left(e^{i\theta_2} - J_0(m)\right)\right)i - \hat{I}\left(e^{i\theta_1} + J_0(m)\right) - \hat{\delta}\nu_1\left(e^{i\theta_1} - J_0(m)\right) \\
&- 2J_1(m)\left(\hat{Q}\cos\Omega t + i\hat{I}\sin\Omega t\right) + 2\hat{\delta}\nu_2 J_1(m)\cos\Omega t + \hat{\delta}\nu_1 J_1(m)\left(e^{i\Omega t} - e^{-i\Omega t}\right)\Bigg] \\
\approx& \frac{1}{2\sqrt{2}}\Bigg[\left(\frac{1}{\sqrt{2}}\left(\gamma + \hat{\delta} b + \hat{\delta}\nu_g\right)\left(e^{i\theta_2} + J_0(m)\right) + \hat{\delta}\nu_2\left(e^{i\theta_2} - J_0(m)\right)\right)i - \\
&\frac{1}{\sqrt{2}}\left(\gamma + \hat{\delta} b - \hat{\delta}\nu_g\right)\left(e^{i\theta_1} + J_0(m)\right) - \hat{\delta}\nu_1\left(e^{i\theta_1} - J_0(m)\right) \\
&- J_1(m)e^{i\Omega t}\left(\frac{2}{\sqrt{2}}\left(\gamma + \hat{\delta} b\right) - \hat{\delta}\nu_1 - \hat{\delta}\nu_2\right) - J_1(m)e^{-i\Omega t}\left(\frac{2}{\sqrt{2}}\hat{\delta}\nu_g + \hat{\delta}\nu_1 - \hat{\delta}\nu_2\right)\Bigg],
\end{aligned} \tag{S10}$$

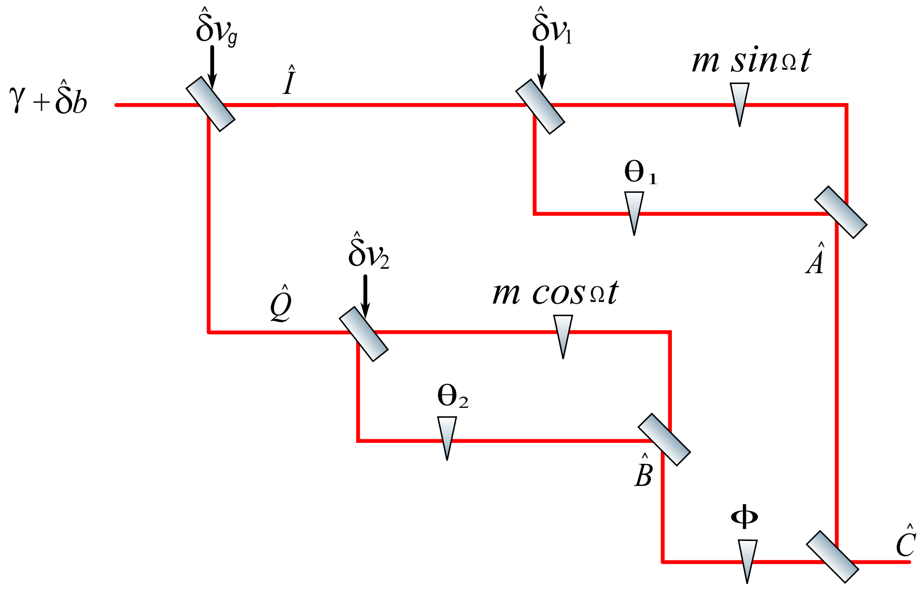

Fig. S2. Schematic of IQ modulator .

where $J_x(m)$ is the n-th Bessel function of the first kind. For a small modulation depth ($m \ll 1$) as in CVQKD system and perfect carrier suppression ($\theta_1 = \theta_2 = \pi$), the output can be simplified as,

$$\hat{C} \approx \frac{1}{2\sqrt{2}}\left[2\left(\hat{\delta}v_1 - i\hat{\delta}v_2\right) - me^{i\Omega t}\left(\frac{2}{\sqrt{2}}\left(\gamma + \hat{\delta b}\right) - \hat{\delta}v_1 - \hat{\delta}v_2\right) - me^{-i\Omega t}\left(\frac{2}{\sqrt{2}}\hat{\delta}v_g + \hat{\delta}v_1 - \hat{\delta}v_2\right)\right]. \tag{S11}$$

Eq. S11 clearly illustrates the complete suppression of the carrier γ, the carrier noise $\delta\hat{b}$ and the negative sideband, depicted by the first and last vacuum terms, respectively. However, the modulation not only transfers photons from the carrier to the modulation sideband but also photons from the RIN as seen from the term $me^{i\Omega t}\delta\hat{b}$. To understand their influence better, we can take the PSD of quadrature operators, for instance, the amplitude quadrature operator ($\hat{X}$),

$$\begin{aligned}\mathrm{PSD}(\hat{X}) = \mathrm{PSD}(\hat{C} + \hat{C}^\dagger) &\approx \frac{1}{2}\left(\mathrm{PSD}(\hat{X}_{\delta v_1}) + \mathrm{PSD}(\hat{P}_{\delta v_2})\right) + \frac{m^2}{4}\left(\gamma^2 + \mathrm{PSD}(\hat{X}_{\delta b}) + \mathrm{PSD}(\hat{X}_{\delta v_g})\right) \\ &\approx \frac{1}{2}\left(\mathrm{PSD}(\hat{X}_{\delta v_1}) + \mathrm{PSD}(\hat{P}_{\delta v_2})\right) + \frac{m^2}{4}\left(\gamma^2 + \mathrm{RIN}\gamma^2 + \mathrm{PSD}(\hat{X}_{\delta v_g})\right),\end{aligned} \tag{S12}$$

where the third term represents the modulation, scaling with laser power, and the 4th term is the RIN contribution. Here, one can note that RIN contribution is in order of -135 dB or less in comparison to the modulation term. Moreover, operating at low modulation depth suppresses the effect of the RIN in the modulated sideband.

Digital-to-analog-converter noise Here, we consider the modulation excess noise caused by the noise associated with the digital-to-analog converter (DAC). To estimate ξ_{DAC} at the receiver side, one can use the following simple equation,

$$\xi_{\mathrm{DAC}} = T\tau V_{\mathrm{mod}}, \tag{S13}$$

where $\tau = 1/\mathrm{SNR}$ is a scaling factor presenting the electrical driving signal of the IQ modulator. To measure τ, we perform two measurements with an electrical spectrum analyzer: without modulation (to obtain the noise floor) and with modulation. The result is shown in Fig. S3. We obtained $\tau \approx -52$ dB. By substituting this value in Eq. S13, $\xi_{\mathrm{DAC}} \approx 1\ \mu$ SNU.

Raman noise The excess noise due to the Spontaneous anti-Stokes Raman scattering (SASRS) from the pilot tone or the optical carrier is given by (*43*),

$$\xi_{\mathrm{Ram}} = \frac{\lambda^3}{hc^2} P_{\mathrm{out}} \beta_{\mathrm{Ram}} L, \tag{S14}$$

where λ is the wavelength, P_{out} is optical power at the receiver of either the pilot tone or the optical carrier, c is the speed of light, β_{Ram} is the spontaneous Raman scattering coefficient and L is the fiber length. Assuming $\beta = 4 \times 10^{-9}$/km nm (*43*), and using Eq. S4 to compute the power of the pilot tone (24.2 pW) and the optical carrier (0.4 pW) at the receiver, we obtain $\xi_{\mathrm{Ram}} \approx 6 \times 10^{-10}$ SNU and 1×10^{-10} SNU for the pilot tone and beat signal, respectively.

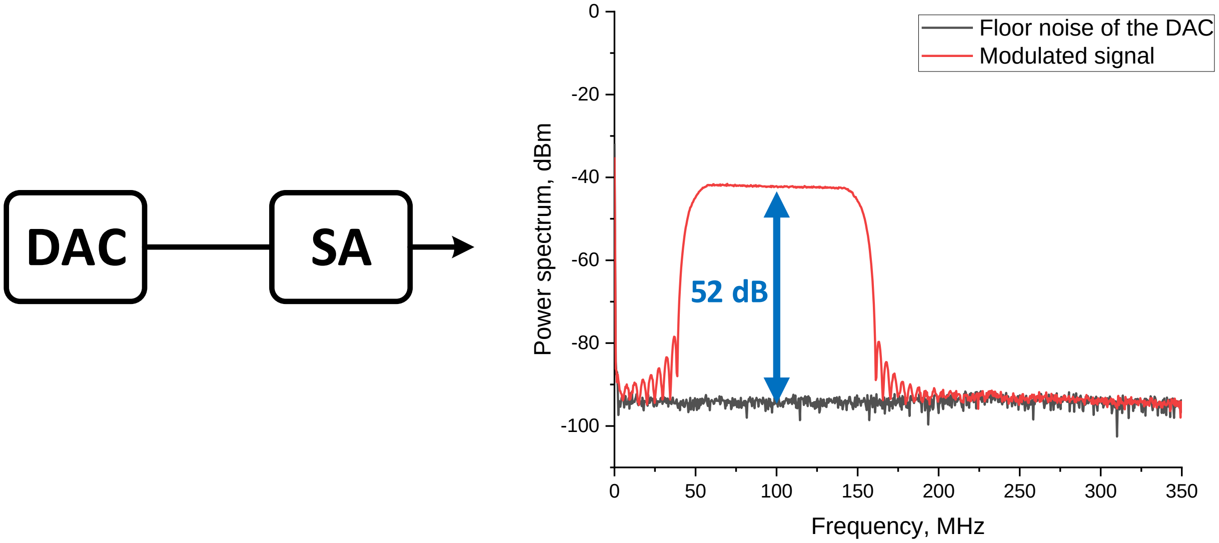

Fig. S3. Schematic of modulation noise characterization. SA: spectrum analyzer; DAC: digital-to-analog converter.

Phase noise As the variance of the residual phase noise (RPN) cannot be directly derived from the measurement, the evaluation of the excess noise due to the RPN is not a trivial task (*44*). To estimate the RPN, we performed a phase noise measurement using the transmitted local oscillator (TLO) setup shown in Fig. S4, where a continuous wave (CW) laser (NKT Photonic Basik E15) was shared between Alice and Bob as the optical signal source and local oscillator (LO). Then, we digitally generated a pilot tone with the phase being modulated with an actual phase profile of the laser $\phi_{\text{modulated}}$. This phase profile of the laser was measured at a very high signal-to-noise ratio (SNR). Next, the digital waveform was uploaded to the DAC to drive the IQ modulator. A variable optical attenuator (VOA) was used to adjust the pilot tone's SNR to the same SNR as used in the long-distance measurement. At the receiver, Bob, a balanced detector flowed by an ADC was deployed to detect and digitize the signal. Finally, we estimated the phase of the pilot tone using an unscented Kalman filter (UKF) and calculated the RPN as,

$$\text{RPN} = \text{Var}(\phi_{\text{estimated}} - \phi_{\text{modulated}}), \tag{S15}$$

where Var denotes the variance and $\phi_{\text{estimated}}$ is the estimated phase. The experimental result was RPN ≈ 0.001 rad^2. Finally, using Eq. 2 in the manuscript with the value above for the RPN, the excess noise ξ_{RPN} was estimated to be 160 μSNU.

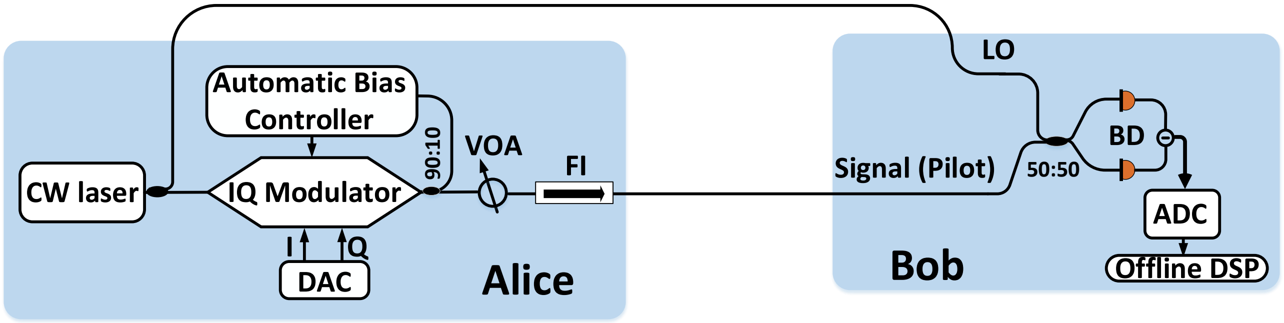

Fig. S4. Experimental setup for phase noise measurement. CW laser: Continuous wave laser; DAC: Digital-to-analog converter; VOA: Variable optical attenuator; FI: Faraday isolator; BD: balanced detector; ADC: Analog-to-digital converter; DSP: Digital signal processing.

S2. System stability

Due to finite-size effects, long measurements are required and hence system stability is very crucial. In particular, the stability of the vacuum noise variance is of utmost importance as the vacuum noise variance is the reference for the estimation of the covariance matrix and there the excess noise and the transmission. A drifting reference could lead to an underestimation of the noise sources and, thus, lead to a security breach.To quantify the stability of the vacuum noise, we considered the Allan deviation, which is a standard method to quantify the stability of a measurement variable. For this purpose, we performed a very long vacuum noise measurement with 5×10^9 symbols, which took 50 s. Fig. S5 shows the (overlapped) Allan deviation as a function of the averaging time. The linear decay of the Allan deviation as the averaging time increases indicates that the system is stable for at least 25 s, which is more than the time required for performing the quantum signal and vacuum measurement. Therefore, our autonomous system can be used to perform the experiment reliably. While it would be interesting to determine the breaking point of the system in terms of the maximum time the system is stable, this was unfortunately not possible due to inefficiencies of the Python code calculating the overlapped Allan deviation.

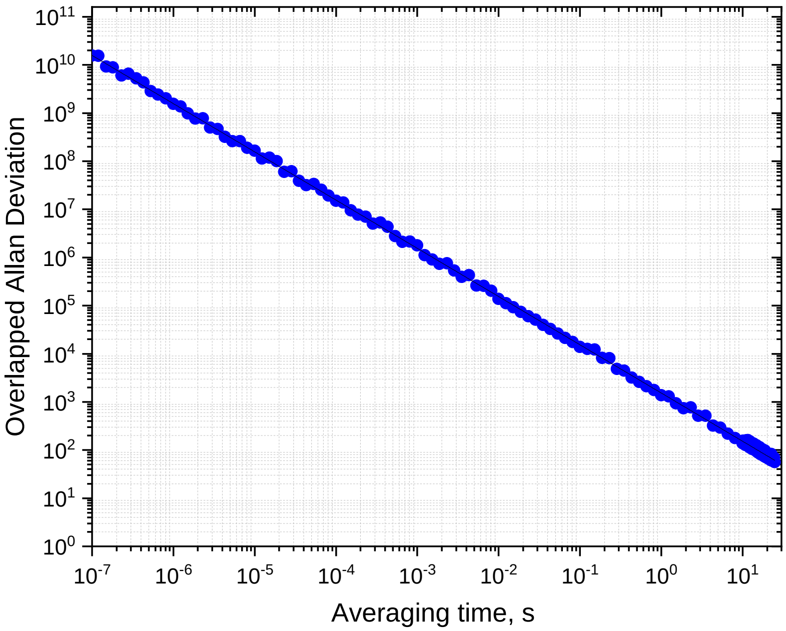

Fig. S5. System stability measurement.

S3. Composable security

In this section, we will analyse the main limiting factors for achieving composable security to provide possible way for improvement. We refer to recent work of Ref. [30] for details on the key rate evaluation for collective attacks in the strongest security context. While the confidence intervals determined during parameter estimation have a major impact on the achievable key rate [30], there are two other terms that are dominant in reducing the composably secure key length: Δ_{AEP} – reflecting the inability to directly apply asymptotic equipartition property to blocks of finite size and the need to employ a weak version of it; and Δ_{ent} – accounting for possible deviation of empirical entropy from true entropy. Both terms are independent of channel parameters and, aside from assigned estimation failure probabilities, are mostly determined by the block size N.

The sum of those penalties is indicated by the shaded red region in Fig. S6. Concurrently, the projected finite-size key lengths, associated with larger block sizes, are depicted by blue lines corresponding to different reconciliation efficiency: the current work's efficiency at $\beta = 92.5\%$, high achievable efficiency at $\beta = 96\%$, and efficiency close to the theoretical limit at $\beta = 99\%$. Using Gaussian finite-size confidence intervals [37], a three-orders-of-magnitude increase in data block size is necessary to achieve a positively composable key without accepting higher failure probabilities while maintaining the current reconciliation efficiency. Enhancing information reconciliation efficiency could potentially relax this requirement by an order of magnitude. Note that the key length in Fig. S6 is evaluated in the finite-size regime with estimation bounds on the channel parameters only, while a comprehensive composable security analysis would impose estimation bounds on all elements of the covariance matrix of the shared states, which might result in a more conservative lower bound on the key. Nevertheless, Fig.S6

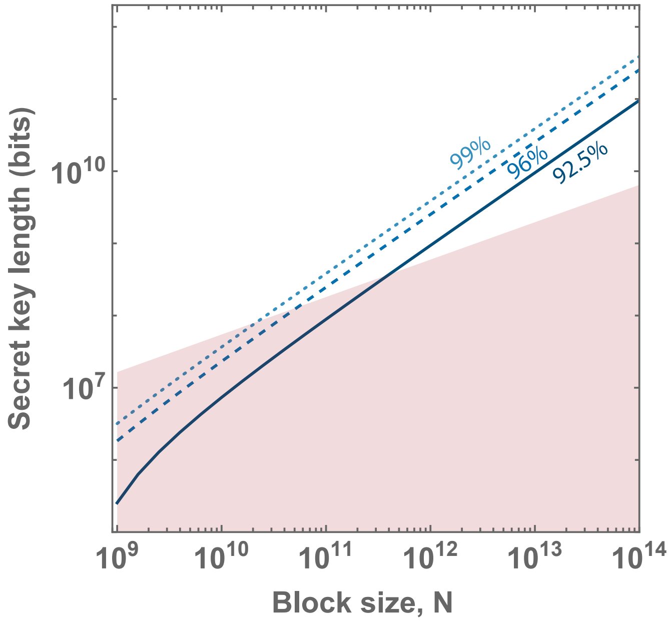

Fig. S6. Composable secret key generation. The block-size dependency of the finite-size key lengths (blue lines) for different reconciliation efficiencies (from bottom to top) $\beta = 92.5\%$, 96%, and 99%). Red region indicates the sum of dominant terms $\Delta_{AEP} + \Delta_{ent}$ in the composable secure key length estimation (see Ref. 30 for details), that needs to be overcome to distill a secure key.

offers valuable insight on the order of magnitude of data volume needed to establish a secure key. Reducing the frame error rate can further alleviate the demand for a larger block size, however, usually just by a small factor.

REFERENCES AND NOTES

1. M. Hellman, New directions in cryptography. *IEEE Trans. Inf. Theory* **22**, 644–654 (1976).

2. R. L. Rivest, A. Shamir, L. Adleman, A method for obtaining digital signatures and public-key cryptosystems. *Commun. ACM* **21**, 120–126 (1978).

3. P. W. Shor, Algorithms for quantum computation: Discrete logarithms and factoring, in *Proceedings 35th Annual Symposium on Foundations of Computer Science* (IEEE, 1994), pp. 124–134.

4. F. Arute, K. Arya, R. Babbush, D. Bacon, J. C. Bardin, R. Barends, R. Biswas, S. Boixo, F. G. S. L. Brandao, D. A. Buell, B. Burkett, Y. Chen, Z. Chen, B. Chiaro, R. Collins, W. Courtney, A. Dunsworth, E. Farhi, B. Foxen, A. Fowler, C. Gidney, M. Giustina, R. Graff, K. Guerin, S. Habegger, M. P. Harrigan, M. J. Hartmann, A. Ho, M. Hoffmann, T. Huang, T. S. Humble, S. V. Isakov, E. Jeffrey, Z. Jiang, D. Kafri, K. Kechedzhi, J. Kelly, P. V. Klimov, S. Knysh, A. Korotkov, F. Kostritsa, D. Landhuis, M. Lindmark, E. Lucero, D. Lyakh, S. Mandrà, J. R. McClean, M. McEwen, A. Megrant, X. Mi, K. Michielsen, M. Mohseni, J. Mutus, O. Naaman, M. Neeley, C. Neill, M. Y. Niu, E. Ostby, A. Petukhov, J. C. Platt, C. Quintana, E. G. Rieffel, P. Roushan, N. C. Rubin, D. Sank, K. J. Satzinger, V. Smelyanskiy, K. J. Sung, M. D. Trevithick, A. Vainsencher, B. Villalonga, T. White, Z Jamie Yao, P. Yeh, A. Zalcman, H. Neven, J. M. Martinis, Quantum supremacy using a programmable superconducting processor. *Nature* **574**, 505–510 (2019).

5. C. H. Bennett, G. Brassard, Quantum cryptography: Public key distribution and coin tossing, in *Proceedings of IEEE International Conference on Computers, Systems, and Signal Processing* (India, 1984), p. 175.

6. S. Pirandola, R. Laurenza, C. Ottaviani, L. Banchi, Fundamental limits of repeaterless quantum communications. *Nat. Commun.* **8**, 15043 (2017).

7. S. Pirandola, U. L. Andersen, L. Banchi, M. Berta, D. Bunandar, R. Colbeck, D. Englund, T. Gehring, C. Lupo, C. Ottaviani, J. L. Pereira, M. Razavi, J. Shamsul Shaari, M. Tomamichel, V. C. Usenko, G. Vallone, P. Villoresi, P. Wallden, Advances in quantum cryptography. *Adv. Opt. Photonics* **12**, 1012–1236 (2020).

8. F. Grosshans, P. Grangier, Continuous variable quantum cryptography using coherent states. *Phys. Rev. Lett.* **88**, 057902 (2002).

9. C. Weedbrook, A. M. Lance, W. P. Bowen, T. Symul, T. C. Ralph, P. Koy, Quantum cryptography without switching. *Phys. Rev. Lett.* **93**, 170504 (2004).

10. J. Lodewyck, T. Debuisschert, R. Tualle-Brouri, P. Grangier, Controlling excess noise in fiber-optics continuous- variable quantum key distribution. *Phys. Rev. A* **72**, 050303 (2005).

11. A. Leverrier, R. Alléaume, J. Boutros, G. Zémor, P. Grangier, Multidimensional reconciliation for a continuous-variable quantum key distribution. *Phys. Rev. A* **77**, 042325 (2008).

12. J. Lodewyck, M. Bloch, R. García-Patrón, S. Fossier, E. Karpov, E. Diamanti, T. Debuisschert, N. J. Cerf, R. Tualle-Brouri, S. W. McLaughlin, P. Grangier, Quantum key distribution over 25 km with an all-fiber continuous-variable system. *Phys. Rev. A* **76**, 042305 (2007).

13. P. Jouguet, S. Kunz-Jacques, A. Leverrier, P. Grangier, E. Diamanti, Experimental demonstration of long-distance continuous-variable quantum key distribution. *Nat. Photonics* **7**, 378–381 (2013).

14. C. Wang, D. Huang, P. Huang, D. Lin, J. Peng, G. Zeng, 25 MHz clock continuous-variable quantum key distribution system over 50 km fiber channel. *Sci. Rep.* **5**, 14607 (2015).

15. D. Huang, P. Huang, D. Lin, G. Zeng, Long-distance continuous-variable quantum key distribution by controlling excess noise. *Sci. Rep.* **6**, 19201 (2016).

16. Y. Zhang, Z. Chen, S. Pirandola, X. Wang, C. Zhou, B. Chu, Y. Zhao, B. Xu, S. Yu, H. Guo, Long-distance continuous-variable quantum key distribution over 202.81 km of fiber. *Phys. Rev. Lett.* **125**, 010502 (2020).

17. X.-C. Ma, S.-H. Sun, M.-S. Jiang, L.-M. Liang, Local oscillator fluctuation opens a loophole for eve in practical continuous-variable quantum-key-distribution systems. *Phys. Rev. A* **88**, 022339 (2013).

18. P. Jouguet, S. Kunz-Jacques, E. Diamanti, Preventing calibration attacks on the local oscillator in continuous- variable quantum key distribution. *Phys. Rev. A* **87**, 062313 (2013).

19. B. Qi, L.-L. Huang, L. Qian, H.-K. Lo, Experimental study on the Gaussian-modulated coherent-state quantum key distribution over standard telecommunication fibers. *Phys. Rev. A* **76**, 052323 (2007).

20. B. Qi, P. Lougovski, R. Pooser, W. Grice, M. Bobrek, Generating the local oscillator "locally" in continuous-variable quantum key distribution based on coherent detection. *Phys. Rev. X* **5**, 041009 (2015).

21. D. Huang, P. Huang, D. Lin, C. Wang, G. Zeng, High-speed continuous-variable quantum key distribution without sending a local oscillator. *Opt. Lett.* **40**, 3695–3698 (2015).

22. S. Kleis, M. Rueckmann, C. G. Schaeffer, Continuous variable quantum key distribution with a real local oscillator using simultaneous pilot signals. *Opt. Lett.* **42**, 1588–1591 (2017).

23. H.-M. Chin, N. Jain, D. Zibar, U. L. Andersen, T. Gehring, Machine learning aided carrier recovery in continuous-variable quantum key distribution. *NPJ Quantum Inf.* **7**, 20 (2021).

24. F. Laudenbach, B. Schrenk, C. Pacher, M. Hentschel, C.-H. F. Fung, F. Karinou, A. Poppe, M. Peev, H. Hübel, Pilot-assisted intradyne reception for high-speed continuous-variable quantum key distribution with true local oscillator. *Quantum* **3**, 193 (2019).

25. A. Marie, R. Alléaume, Self-coherent phase reference sharing for continuous-variable quantum key distribution. *Phys. Rev. A* **95**, 012316 (2017).

26. Y. Pi, H. Wang, Y. Pan, Y. Shao, Y. Li, J. Yang, Y. Zhang, W. Huang, B. Xu, Sub-Mbps key-rate continuous-variable quantum key distribution with local local oscillator over 100-km fiber. *Opt. Lett.* **48**, 1766–1769 (2023).

27. L. Li, T. Wang, X. Li, P. Huang, Y. Guo, L. Lu, L. Zhou, G. Continuous-variable quantum key distribution with on-chip light sources. *Photonics Res.* **11**, 504–516 (2023).

28. V. Scarani, H. Bechmann-Pasquinucci, N. J. Cerf, M. Dusek, N. Lutkenhaus, M. Peev, The security of practical quantum key distribution. *Rev. Mod. Phys.* **81**, 1301 (2009).

29. F. Laudenbach, C. Pacher, C.-H. F. Fung, A. Poppe, M. Peev, B. Schrenk, M. Hentschel, P. Walther, H. Hübel, Continuous-variable quantum key distribution with Gaussian modulation—The theory of practical implementations. *Adv. Quantum Technol.* **1**, 1800011 (2018).

30. N. Jain, H.-M. Chin, H. Mani, C. Lupo, D. S. Nikolic, A. Kordts, S. Pirandola, T. B. Pedersen, M. Kolb, B. Ömer, C. Pacher, T. Gehring, U. L. Andersen, Practical continuous-variable quantum key distribution with composable security. *Nat. Commun.* **13**, 4740 (2022).

31. H. Mani, T. Gehring, P. Grabenweger, B. Ömer, C. Pacher, U. L. Andersen, Multiedge-type low-density parity-check codes for continuous-variable quantum key distribution. *Phys. Rev. A* **103**, 062419 (2021).

32. A. A. Hajomer, N. Jain, H. Mani, H.-M. Chin, U. L. Andersen, T. Gehring, Modulation leakage-free continuous-variable quantum key distribution. *NPJ Quantum Inf.* **8**, 136 (2022).

33. J. Martinez-Mateo, D. Elkouss, V. Martin, Blind reconciliation. *Quantum Inf. & Comput.* **12**, 791–812 (2012).

34. B.-Y. Tang, B. Liu, Y.-P. Zhai, C.-Q. Wu, W.-R. Yu, High-speed and large-scale privacy amplification scheme for quantum key distribution. *Sci. Rep.* **9**, 15733 (2019).

35. P. Jouguet, S. Kunz-Jacques, E. Diamanti, A. Leverrier, Analysis of imperfections in practical continuous-variable quantum key distribution. *Phys. Rev. A* **86**, 032309 (2012).

36. I. Devetak, A. Winter, Distillation of secret key and entanglement from quantum states. *Proc. R. Soc. A Math. Phys. Eng. Sci.* **461**, 207–235 (2005).

37. L. Ruppert, V. C. Usenko, R. Filip, Long-distance continuous-variable quantum key distribution with efficient channel estimation. *Phys. Rev. A* **90**, 062310 (2014).

38. A. Leverrier, F. Grosshans, P. Grangier, Finite-size analysis of a continuous-variable quantum key distribution. *Phys. Rev. A* **81**, 062343 (2010).

39. A. A. Hajomer, H. Mani, N. Jain, H.-M. Chin, U. L. Andersen, T. Gehring, Continuous-variable quantum key distribution over 60 km optical fiber with real local oscillator, in *European Conference and Exhibition on Optical Communication* (Optica Publishing Group, 2022), pp. Th1G–5.

40. C. Bruynsteen, M. Vanhoecke, J. Bauwelinck, X. Yin, Integrated balanced homodyne photonic–electronic detector for beyond 20 GHz shot-noise-limited measurements. *Optica* **8**, 1146–1152 (2021).

41. Y. Li, X. Zhang, Y. Li, B. Xu, L. Ma, J. Yang, W. Huang, High-throughput GPU layered decoder of quasi-cyclic multi-edge type low density parity check codes in continuous-variable quantum key distribution systems. *Sci. Rep.* **10**, 14561 (2020).

42. T. Gehring, C. Lupo, A. Kordts, D. Solar Nikolic, N. Jain, T. Rydberg, T. B. Pedersen, S. Pirandola, U. L. Andersen, Homodyne-based quantum random number generator at 2.9 Gbps secure against quantum side-information. *Nat. Commun.* **12**, 605 (2021).
43. B. Qi, W. Zhu, L. Qian, H.-K. Lo, Feasibility of quantum key distribution through a dense wavelength division multiplexing network. *New J. Phys.* **12**, 103042 (2010).
44. H.-M. Chin, N. Jain, U. L. Andersen, T. Gehring, Towards optimum phase noise compensation for CV-QKD systems, in *CLEO: Fundamental Science* (Optica Publishing Group, 2023), pp. FF2A–4.
